# Zahnmedizinische Versorgung älterer Menschen: Chancen und Herausforderungen

**DOI:** 10.1007/s00103-021-03358-1

**Published:** 2021-06-22

**Authors:** Ina Nitschke, Sebastian Hahnel

**Affiliations:** 1 Poliklinik für Zahnärztliche Prothetik und Werkstoffkunde, Universitätsklinikum Leipzig, Universität Leipzig, Liebigstraße 12, Haus 1, 04103 Leipzig, Deutschland; 2 Klinik für Allgemein‑, Behinderten- und Seniorenzahnmedizin, Universität Zürich, Zürich, Schweiz

**Keywords:** Mundgesundheit, Geriatrische Zahnmedizin, Menschen mit Pflegebedarf, Chancengleicher Zugang, Inanspruchnahmeverhalten, Oral health, Geriatric dentistry, People with care requirements, Equal opportunities, Take-up of dental services

## Abstract

Die Mundgesundheit der Bevölkerung in Deutschland konnte in den letzten Jahren verbessert werden; allerdings profitierten ältere und insbesondere gebrechliche sowie pflegebedürftige Menschen nicht adäquat von dieser Entwicklung. Dabei kann eine gute Mundgesundheit relevant dazu beitragen, die Herausforderungen bei Gebrechlichkeit und Pflegebedürftigkeit besser zu bewältigen. Der eingeschränkte Zugang zur zahnmedizinischen Betreuung, die manchmal eingeschränkte Kooperativität sowie die schlechtere Mundpflege in dieser Bevölkerungsgruppe erhöhen das Risiko für Karies, Parodontalerkrankungen, Zahnverlust und Zahnlosigkeit im Vergleich zur Durchschnittsbevölkerung.

Der vorliegende Beitrag gibt eine Übersicht über die zahnmedizinische Situation älterer Menschen anhand bereits publizierter Daten aus der bevölkerungsrepräsentativen Fünften Deutschen Mundgesundheitsstudie (DMS V), die im Jahr 2014 erhoben wurden. Die mittlere Anzahl der fehlenden Zähne betrug bei den 65- bis 74-Jährigen 11,1 Zähne. Bei älteren Seniorinnen und Senioren (75–100 Jahre) mit Pflegebedarf (äSmP) war die Mundgesundheit schlechter als bei denjenigen ohne Pflegebedarf (äSoP). So hatten äSoP durchschnittlich 11,8 Zähne, äSmP dagegen nur 5,7 Zähne. Der Anteil zahnloser 65- bis 74-Jähriger hatte sich seit 1997 halbiert auf 12,4 %. Bei den äS waren 32,8 % zahnlos (äSmP: 53,7 %, äSoP: 26,7 %). Mehr als 75 % der äSmP waren abnehmbar prothetisch versorgt (äSoP: 51,7 %). Vor diesem Hintergrund werden in diesem Beitrag Schnittstellen benannt, an denen eine chancengleiche Anbindung dieser Bevölkerungsgruppen an eine zahnmedizinische Versorgungsstruktur etabliert werden könnte. Diese beinhalten akutgeriatrische Krankenhausstationen und die Entwicklung weitergehender Konzepte in der aufsuchenden Versorgung zur besseren Versorgung der Betroffenen und zur Erleichterung der Pflege.

## Abstract (en)

The oral health of the population in Germany has improved in recent years; however, older people, especially those with frailty and in need of care, have not benefited adequately from this development. Yet, good oral health can make a relevant contribution to better coping with the challenges of frailty and the need for long-term care. Limited access to dental care, and in some cases reduced cooperation and poorer oral hygiene, increases the risk of caries, periodontal diseases, and loss of teeth as well as leads to a higher prevalence of edentulism in comparison to the general population.

This paper provides an overview of the dental situation of older people based on previously published data from the population representative Fifth German Oral Health Study (DMS V), which was collected in 2014. The mean number of missing teeth was 11.1 among 65- to 74-year-olds. Older seniors (75–100 years) with care needs (äSmP) had worse oral health than those without care needs (äSoP). Thus, on average, äSoP had 11.8 teeth, whereas äSmP had only 5.7 teeth. The proportion of edentulous 65–74-year-olds had halved since 1997 to 12.4%. Among the äS, 32.8% were edentulous (äSmP: 53.7%, äSoP: 26.7%). More than 75% of the äSmP had removable prosthetic restorations (äSoP: 51.7%). Against this background, this paper identifies interfaces where an equal opportunity connection of these population groups to a dental care structure could be established. These include acute geriatric hospital wards and the development of more far-reaching concepts in outreach care to improve care for those affected and to facilitate care.

## Einleitung

Der Anteil älterer Menschen an der Gesamtbevölkerung ist steigend [1], sodass sich die sozialen Versorgungssysteme immer größeren Herausforderungen stellen müssen. In Deutschland haben alle Bürgerinnen und Bürger das Recht auf eine bedarfsgerechte gesundheitliche Versorgung. Dieses Recht ist Bestandteil der Menschenrechte (UN-Behindertenrechtskonvention, 26.03.2009 für Deutschland ratifiziert [2]), welche sicherstellen, dass das individuell erreichbare Höchstmaß an Gesundheit ohne Diskriminierung aufgrund einer Behinderung zur Verfügung steht. Hier sind auch Ältere sowie Kinder, Gebrechliche und Pflegebedürftige eingeschlossen. Eine bedarfsgerechte gesundheitliche Versorgung beinhaltet die gesundheitliche Prävention, Behandlung und Betreuung. Die ambulanten und stationären Versorgungssysteme sind bei nachlassender funktioneller Kapazität innerhalb der heterogenen Bevölkerungsgruppe der Seniorinnen und Senioren gefordert Versorgungsangebote bereitzuhalten, die den Notwendigkeiten der sich stetig und manchmal sehr schnell ändernden Ansprüche angepasst sind.

In dieses Versorgungssystem, welches durch die politischen Organe der Gesetzgebung in der Makroebene definiert, durch die Krankenversicherer in der Mesoebene umgesetzt und durch die im Gesundheitssystem Beschäftigten in der Mikroebene ausgeführt wird, gehört auch die Behandlung oraler Erkrankungen. Die Mundgesundheit ist durch präventive Maßnahmen zu schützen oder wiederherzustellen, wenn Defizite auftreten.

Durch Präventionsmaßnahmen verfügen ältere Menschen über deutlich mehr Zähne als noch vor 20 Jahren; Zahnlosigkeit ist seltener geworden und Zahnersatz kann auf hohem Qualitätsniveau hergestellt werden. Dies ist jedoch nur möglich, wenn nach einer partizipativen Therapieentscheidung eine Behandlung bei entsprechend belastbaren und kooperativen Seniorinnen und Senioren erfolgt. Diese Voraussetzungen findet die Zahnärztin oder der Zahnarzt in der Regel bei den funktionell nicht eingeschränkten fitten älteren Menschen. Bei gebrechlichen oder pflegebedürftigen Seniorinnen und Senioren präsentieren sich jedoch andere Herausforderungen, da die Folgen körperlicher und kognitiver Einschränkungen die zur Verfügung stehenden Therapiemöglichkeiten stark limitieren. Im Gegensatz zu Seniorinnen und Senioren ohne Pflegebedarf (SoP) weisen Seniorinnen und Senioren mit Pflegebedarf (SmP) eine schlechtere Mundgesundheit auf [3–5]. Daher sind auch andere Personen – Angehörige, gesetzliche Betreuende, Pflegepersonal, Ärztinnen und Ärzte – zunehmend in die zahnmedizinische Versorgung einzubeziehen. Das Gesundheitswesen ist gefordert, auch bei schwierigen Lebensumständen eine bestmögliche Mundgesundheit zu ermöglichen. Eine gute Mundgesundheit, verbunden mit infektionsfreier Mundhöhle und passendem Zahnersatz, trägt zur Lebensqualität, Vermeidung von allgemeinmedizinischen Krankheiten und Komplikationen bei Seniorinnen und Senioren bei.

In dem Beitrag wird die Heterogenität der älteren Menschen hinsichtlich ihrer Mundgesundheit beschrieben und die sich daraus ergebenden Problemfelder für eine zahnmedizinische Betreuung werden dargestellt. Die Differenzen innerhalb der Mundgesundheit werden an zahnmedizinischen Daten einer bevölkerungsrepräsentativen Studie für Menschen mit und ohne Pflegbedarf erläutert. Verschiedene strukturelle Probleme werden benannt und Lösungsvorschläge gemacht, um chancengleiche Anbindung an eine zahnmedizinische Versorgungsstruktur zu etablieren.

## Lebensphasen als Herausforderung

Der Alterungsprozess gestaltet sich mit dem Auftreten von chronischen Erkrankungen sehr unterschiedlich. Dabei sind 3 Phasen zu differenzieren, wobei sich an die erste fitte und zweite gebrechliche Phase oftmals eine dritte pflegebedürftige Lebensphase anschließt. Relevant sind in diesem Kontext insbesondere die Übergänge – also von fit zu gebrechlich sowie von gebrechlich zu pflegebedürftig. Oftmals durchlaufen Seniorinnen und Senioren alle 3 Phasen, wobei sie unterschiedliche Unterstützungssysteme benötigen. Hierbei ist nicht allein die Versorgung innerhalb der jeweiligen Phase relevant. Vielmehr besteht die Herausforderung darin, den Übergang in die nächste Phase möglichst hinauszuzögern und gut vorzubereiten. Dabei ist es die tägliche Herausforderung der Zahnmedizin und anderer im Gesundheitswesen tätiger Akteure, die Mundgesundheit innerhalb dieser Lebensphasen aufrechtzuerhalten.

## Mundgesundheit älterer Menschen mit und ohne Pflegebedarf

Die im Jahr 2016 veröffentlichte Fünfte Deutsche Mundgesundheitsstudie (DMS V; [6]) hat 2014 2 Gruppen der Seniorinnen und Senioren einbezogen (jüngere der Altersgruppe 65–74 Jahre, *n* = 1042, und ältere der Altersgruppe 75–100 Jahre, *n* = 1133). In dieser für Deutschland repräsentativen epidemiologischen Erhebung wurden in der Gruppe der älteren Seniorinnen und Senioren (äS), der Bevölkerung entsprechend, auch ältere Senioren mit Pflegebedarf (äSmP *N* = 256) in die Studie aufgenommen [5]. Bei der Interpretation der Daten ist zu berücksichtigen, dass sowohl Menschen mit ausgeprägter Demenz als auch Menschen in einer Palliativsituation nicht in die Studie eingeschlossen wurden. Die Daten der äSmP innerhalb DMS V [5] dürften somit die Realität etwas beschönigen. Auch sind Aussagen mit Vergleichen zwischen den DMS III, IV und V durch die zeitlich aufeinander aufbauenden Querschnittsstudien limitiert.

### Erkrankungen der Mundschleimhaut

Prothesenbedingte Mundschleimhautveränderungen (z. B. Druckstellen, Ulzerationen, Reizfibrome) treten bei den äSmP (6,9 %) im Vergleich zu älteren Senioren ohne Pflegebedarf (äSoP) fast doppelt so häufig auf. Bei äSmP wurde 7‑mal häufiger als bei äSoP ein oraler Lichen planus diagnostiziert, wobei hier die insgesamt geringen Fallzahlen berücksichtigt werden sollten ([5]; Tab. 1).

**Table 1:** 

	Ohne Pflegestufe	Mit Pflegestufe	Gesamt
**Mundschleimhautveränderungen**	*n* = 877	*n* = 256	*n* = 1133
Karzinom (%)	0,2	0,0	0,2
Leukoplakie (%)	0,8	0,5	0,8
Erythroplakie (%)	0,6	1,0	0,7
Oraler Lichen planus (%)	0,1	0,7	0,2
Candida (%)	0,1	0,0	0,1
Raucherkeratose (%)	0,2	0,0	0,2
Prothesenbedingte Veränderung (%)	3,7	6,9	4,4
Sonstiges (%)	9,8	3,6	8,4
**Parodontale Erkrankungen** (basierend auf Full Mouth Protocol)	*n* = 642	*n* = 119	*n* = 761
BOP (%)	43,2	64,3	46,5
Mittlere ST (mm)	2,8	2,8	2,8
Mittlerer AV (mm)	3,9	4,4	4,0
*CPI*	*n* = 611	*n* = 111	*n* = 722
Grad 0, 1, 2 (%)	17,5	30,0	19,4
Grad 3 (ST 4–5 mm; %)	53,3	35,1	50,5
Grad 4 (ST ≥ 6 mm; %)	29,2	34,9	30,1
*CDC/AAP Fallklassifikation*	*n* = 544	*n* = 96	*n* = 640
Keine/milde Parodontitis (%)	8,5	18,3	10,0
Moderate Parodontitis (%)	47,7	34,1	45,7
Schwere Parodontitis (%)	43,7	47,6	44,3
**Anzahl fehlender Zähne**	*n* = 877	*n* = 256	*n* = 1133
Ohne Weisheitszähne	16,5	22,4	17,8
Mit Weisheitszähnen	20,2	26,3	21,6
**Zahnlosigkeit**	*n* = 877	*n* = 256	*n* = 1133
Ober- und Unterkiefer (%)	26,7	53,7	32,8
Oberkiefer (%)	42,3	63,6	47,1
Unterkiefer (%)	28,1	55,8	34,4
**Art des Zahnersatzes**	*n* = 877	*n* = 256	*n* = 1133
Kein Zahnersatz (%)	8,1	5,0	7,4
Abnehmbar (%)	51,7	77,1	57,4
Festsitzend (%)	23,1	8,3	19,8
Abnehmbar und festsitzend (%)	17,1	9,6	15,4
**Inanspruchnahmeverhalten**	*n* = 845	*n* = 254	*n* = 1099
Beschwerdeorientiert (%)	31,8	61,3	38,4
Kontrollorientiert (%)	68,2	38,7	61,6

*AV* Attachmentverlust (Verlust an Zahnhalteapparat; Sondierungstiefe plus Ausmaß des Zahnfleischrückganges), *BOP* „bleeding on probing“ (Angaben zum Zahnfleischbluten beim Sondieren), *CDC/AAP-Fallklassifikation* Fallklassifikation der Centers for Disease Control and Prevention/American Academy of Periodontology, *CPI* Community Periodontal Index

### Parodontale Erkrankungen

Keine bzw. eine milde Parodontitis trat bei 18,3 % der äSmP auf und damit mehr als doppelt so häufig wie bei äSoP (8,5 %). Der gingivale Entzündungszustand („bleeding on probing“) war bei den äSmP deutlich höher als bei äSoP. Trotz verringerter Zahnzahl bei den äSmP trat an 64,3 % der untersuchten Stellen eine Blutung auf; hingegen wurde eine Entzündung der Gingiva bei den äSoP nur an 43,2 % der untersuchten Stellen registriert ([5]; Tab. 1).

### Zahnverlust und Zahnersatz

Die mittlere Anzahl der fehlenden Zähne betrug bei den 65- bis 74-Jährigen in den Jahren 1997, 2005 und 2014 jeweils 17,6 Zähne [7], 14,2 Zähne [8] und 11,1 Zähne ([9]; bezogen auf 28 Zähne). ÄSoP haben durchschnittlich 11,8 Zähne, äSmP hingegen nur noch 5,7 Zähne. ÄSmP fehlen unter Berücksichtigung der Weisheitszähne durchschnittlich 26,3 Zähne (ohne Weisheitszähne: 22,4 Zähne) und damit 6,1 Zähne (ohne Weisheitszähne: 5,9 Zähne) mehr als bei äSoP ([5]; Tab. 1).

Durch den Rückgang des Zahnverlustes hat sich der Anteil zahnloser jüngerer Seniorinnen und Senioren seit 1997 [7] von 24,8 %, etwas über 22,6 % im Jahr 2005 [8] bis hin zu 12,4 % im Jahr 2014 [9] halbiert. 32,8 % aller äS sind zahnlos ([10]; äSmP: 53,7 %, äSoP: 26,7 %; [5]). Mehr als 3 Viertel der äSmP sind abnehmbar prothetisch versorgt (äSoP: 51,7 %; Tab. 1).

Zusammenfassend ist festzustellen, dass äSmP eine schlechtere Mundgesundheit als äSoP aufweisen [5]. Daher ist es notwendig, die zahnmedizinische Betreuung für die ambulant und stationär Pflegedürftigen zu verbessern.

## Oralgeriatrisches Assessment bei Gebrechlichkeit oder Pflegebedürftigkeit

Im Jahr 2019 meldeten die Pflegekassen in Deutschland 4.251.638 Menschen mit Pflegebedürftigkeit (davon 3.141.471 ambulant pflegebedürftig; [11]). Das Statistische Bundesamt verweist auf 4.127.605 Pflegebedürftige, davon sind 62,3 % Frauen und 3.309.288 ambulant pflegebedürftig [12]. Es ist im Gegensatz zu den allgemeinmedizinisch Gesunden eine Herausforderung, ältere Menschen mit Multimorbidität und Polymedikation zahnärztlich zu behandeln. Vergleichbar mit Assessments in der Geriatrie sollte es auch in der Seniorenzahnmedizin Standard werden, den älteren Menschen unter zahnmedizinischen Aspekten einzuschätzen, die zahnmedizinische Belastbarkeit festzulegen und diese zu Beginn einer neuen Behandlungsmaßnahme zu reevaluieren. Als oralgeriatrisches Assessmentelement steht dabei die zahnmedizinische funktionelle Kapazität (ZFK) mit den Parametern Therapiefähigkeit, Mundhygienefähigkeit und Eigenverantwortlichkeit zur Verfügung [13–15]. Jeder einzelne Parameter wird nach dem Anamnesegespräch mithilfe der benannten Kriterien von zahnärztlicher Seite bewertet (Tab. 2). Innerhalb der 3 Parameter werden die Patientin oder der Patient in einer 4‑stufigen Einteilung hinsichtlich der Belastbarkeit betrachtet. Dabei ist Stufe 1 die beste (normale Belastbarkeit) und Stufe 4 die schlechteste (keine Belastbarkeit). Beim Parameter Eigenverantwortlichkeit erfolgt eine Einteilung nur in 3 Stufen: normal (Stufe 1), reduziert (Stufe 3) oder gar nicht eigenverantwortlich (Stufe 4; Tab. 3). Für den Gesamtscore, also die Festlegung der Belastbarkeitsstufe (BS) der Patientin oder des Patienten (BS 1, BS 2, BS 3, BS 4), ist der am schlechtesten bewertete Parameter ausschlaggebend.

**Table 2:** 

Parameter der ZFK	Kriterien zur individuellen Einschätzung
Therapiefähigkeit	Behandlungsort
	Transportfähigkeit in zahnärztliche Praxis
	Umsetzen in den Behandlungsstuhl möglich
	Lagerungseinschränkung
	Möglichkeit der Diagnostik
	Längere Mundöffnungsphasen
	Risiko für allgemeinmedizinische Zwischenfälle
	Risiko für Medikamenteninteraktion
	Risiken während der Behandlung
	Verständnis von Anweisungen/Wiedergabe von Sachinhalten
	Nachsorgekompetenz
	Manuelle Geschicklichkeit
	Adaptationsfähigkeit
Mundhygienefähigkeit	Greiffähigkeit
	Handkraft
	Manuelle Geschicklichkeit bei der Durchführung der Mundhygiene
	Sehvermögen
	Durchführung der häuslichen Mundhygiene
	Schwierigkeitsgrad der Reinigbarkeit der Mundhöhle
	Verstehen von Anweisungen und Ratschlägen
	Erhaltene Ratschläge umsetzen
	Nachsorgekompetenz
	Fremdputzer vorhanden zur Überwachung und Durchführung der Mundhygiene
	Fähigkeit und Möglichkeit, Mundhygieneprodukte einzukaufen bzw. zu erhalten
Eigenverantwortlichkeit	Erkennen von Problemen
	Willensäußerung
	Entscheidungsfähigkeit
	Besuchsverhalten
	Organisationsfähigkeit
	Nachsorgekompetenz
	Verantwortungsträger (Vollmacht, Patientenverfügung)
	Gerichtlich eingesetzte Betreuung (Deutschland)/Beistand (Schweiz)

**Table 3:** 

Belastbarkeitsstufe (BS)	Therapiefähigkeit	Mundhygienefähigkeit	Eigenverantwortlichkeit
1	Normal	Normal	Normal
2	Leicht reduziert	Leicht reduziert	
3	Stark reduziert	Stark reduziert	Reduziert
4	Keine	Keine	Keine

Diese BS ist Ausdruck der ZFK. Dieses oralgeriatrische Assessment hilft Zahnärztinnen und Zahnärzten im partizipativen Therapieentscheidungsprozess und der zahnmedizinischen Betreuung, da die Belastbarkeit die begrenzende Komponente ist. Dabei besteht die Herausforderung darin, abzuschätzen, wie stark und in welchem Zeitraum sich die Belastbarkeit der zu Behandelnden verändern wird. Zudem können spezielle Behandlungsverfahren, wie z. B. das Duplizieren von Prothesen oder eine Planung von Zahnersatz nach dem g3-S-Prinzip (simpel, stabil und solide), abgeleitet werden [15].

Die DMS V konnte zeigen, dass 90,2 % der äSoP und 47,8 % der äSmP normal oder leicht reduziert therapiefähig waren. Somit sind fast die Hälfte der äSmP unter zahnmedizinischen Aspekten gut zu therapieren, ein Sachverhalt, der die Heterogenität der Pflegebedürftigen deutlich unterstreicht. Bei den äSmP ist nur etwa jeder Fünfte (22,5 %) in der Lage, eine normale Mundhygiene durchzuführen (äSoP: 63,1 %). Außerdem waren 17,4 % der äSmP nicht mehr in der Lage, eigenverantwortlich Therapieentscheidungen zu treffen oder eigenständig einen Zahnarzttermin zu organisieren bzw. wahrzunehmen (äSoP: 0 %; Tab. 4). An dieser Stelle sei noch einmal darauf hingewiesen, dass Menschen mit intensivem Pflegebedarf aus der Studie ausgeschlossen waren. BS 3 und 4 werden dementsprechend tatsächlich einen höheren Anteil bei den äSmP im Alltag ausmachen [16].

**Table 4:** 

	Ohne Pflegestufe	Mit Pflegestufe	Gesamt
*n*	877	256	1133
*Therapiefähigkeit (%)*			
Normal	64,6	26,1	55,9
Leicht reduziert	25,6	21,7	24,7
Stark reduziert	9,8	43,7	17,5
Keine	0,0	8,7	1,9
*Mundhygienefähigkeit (%)*			
Normal	63,1	22,5	53,9
Leicht reduziert	31,7	34,9	32,4
Stark reduziert	5,3	33,2	11,6
Keine	0,0	9,4	2,1
*Eigenverantwortlichkeit (%)*			
Normal	88,7	41,0	77,9
Reduziert	11,3	41,5	18,2
Keine	0,0	17,4	3,9
*Belastbarkeitsstufe (BS; %)*			
BS 1 – voll belastbar	56,4	17,5	47,6
BS 2 – leicht reduziert	27,6	15,7	24,9
BS 3 – stark reduziert	16,0	48,9	23,4
BS 4 – nicht belastbar	0,0	17,9	4,0

## Zahnärztliche Inanspruchnahme

### Inanspruchnahmeverhalten zahnmedizinischer Leistungen

**Figure 1:**
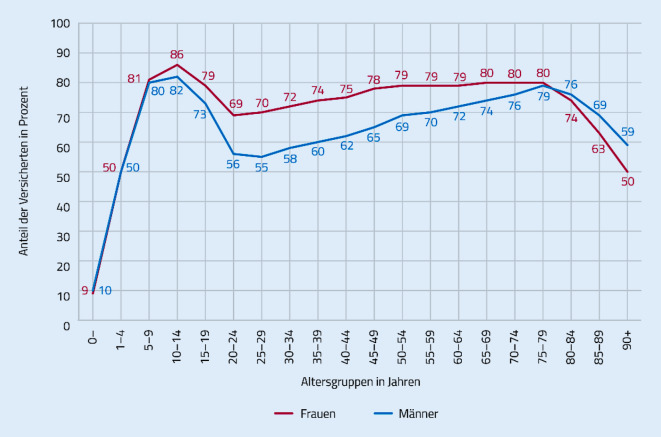

Ärztliche Leistungen werden von fast allen Seniorinnen und Senioren in Anspruch genommen. Dahingegen sinkt die Inanspruchnahme der zahnärztlichen Leistungen: bei den Frauen von zunächst noch 80 % im Alter von 75 bis 79 Jahren auf 50 % ab einem Alter von 90 Jahren. 79 % der Männer im Alter von 75 bis 79 Jahren nehmen zahnärztliche Leistungen in Anspruch, ab einem Alter von 90 Jahren nur noch 59 % ([17]; Abb. 1).

Mit zunehmender Gebrechlichkeit werden Zahnärztinnen oder Zahnärzte oftmals nur noch bei Beschwerden aufgesucht und der präventive Aspekt einer zahnmedizinischen Betreuung geht verloren [18, 19]. Den ambulant Pflegebedürftigen steht seltener als den allgemeinmedizinisch Gesunden ein chancengleicher Zugang zur zahnärztlichen Versorgung zur Verfügung [20]. Die Mundgesundheit tritt zunehmend in den Hintergrund [21–23], wodurch sich längerfristig der zahnärztliche Behandlungsbedarf erhöht. Zudem ist das Inanspruchnahmeverhalten zahnärztlicher Leistungen abhängig vom Vorhandensein einer Pflegestufe: Mehr als 2 Drittel (68,2 %) der äSoP gaben an, die Zahnärztin oder den Zahnarzt kontrollorientiert in Anspruch zu nehmen, wohingegen 61,2 % der äSmP die Praxis nur beschwerdeorientiert aufsuchten ([5]; Tab. 1).

Das Inanspruchnahmeverhalten von medizinischen Dienstleistungen wird durch verschiedene Faktoren geprägt. Der Zugang für Seniorinnen und Senioren zu ärztlichen und zahnärztlichen Leistungen stellt für die Sicherstellung von Chancengleichheit und Gerechtigkeit Ärztinnen und Ärzte sowie Zahnärztinnen und Zahnärzte gleichermaßen vor Herausforderungen und ethische Dilemmata [19, 24]. Im Bereich der Seniorenzahnmedizin lassen sich Muster und Wirkungszusammenhänge von veränderbaren und unveränderbaren (Einfluss‑)Faktoren, Ereignissen (z. B. Krankenhausaufnahme, Verlust naher Angehöriger, Übergang in eine Pflegesituation) und Lebensumständen (z. B. Abnahme der Eigenverantwortlichkeit, reduzierte Mobilität, steigende Morbidität, Armut), welche die Inanspruchnahme reduzieren oder sogar verhindern können, erkennen. Eine Herausforderung für die Akteure im Gesundheitswesen wird es zukünftig sein, sowohl in der Makro- als auch in der Mesoebene des Gesundheitswesens die Voraussetzungen dafür zu schaffen, dass auf der Mikroebene auch die heterogene Gruppe der älteren Menschen zahnärztlich einwandfrei und individuell versorgt werden kann. Beispielhaft seien ein vereinfachter Zugang zur Parodontaltherapie oder die Verordnung einer hochfluoridhaltigen Medikation für SmP zulasten der gesetzlichen Krankenkasse [25] genannt.

### Aufsuchende zahnmedizinische Betreuung

In Deutschland sind unterschiedliche aufsuchende zahnmedizinische Versorgungskonzepte etabliert; diese reichen z. B. von einem einfachen Hausbesuch bei einem langjährigen Patienten bis hin zur vollstrukturierten Praxis mit mehreren Behandlerteams, die täglich SmP aufsuchen (Tab. 5). Neben Eingangsuntersuchung und Therapieplanung besteht die Aufgabe der aufsuchenden Betreuung darin, die weiteren Behandlungsumstände (z. B. medizinische Diagnosen, Medikation, Vorhandensein von Angehörigen) und den Behandlungsort zu klären. Im Allgemeinen ist zu empfehlen, das Praxiskonzept strukturiert wachsen zu lassen. Auf diese Weise kann festgestellt werden, wie sich die Einflussfaktoren (z. B. Haltung der Leitungsebene zur Relevanz der Mundgesundheit von Bewohnerinnen und Bewohnern oder seniorenzugewandte Gesinnung der Mitarbeitenden in der Praxis) entwickeln und ob ein gemeinsames Wachsen an der Aufgabe möglich ist. Dabei sollte das gesamte Team Freude an der Betreuung der oft sehr dankbaren SmP empfinden. Neue Gesetze (GKV-Versorgungsstrukturgesetz (GKV-VStG, in Kraft getreten 2012; [26]), Pflege-Neuausrichtungs-Gesetz (PNG, in Kraft getreten 2013; [27]) und GKV-Versorgungsstärkungsgesetz (GKV-VSG, in Kraft getreten 2015; [28])) bewirken eine bessere Honorierung des Aufwands bei der Versorgung von SmP und sind als Chance für die Betroffenen und die beteiligten Zahnmedizinerinnen und -mediziner zu verstehen. Weiterentwicklungen, z. B. auf Grundlage des A‑ und B‑Konzeptes (Konzept zur vertragszahnärztlichen Versorgung von Pflegebedürftigen und Menschen mit Behinderungen) der wissenschaftlichen Fachverbände und der zahnmedizinischen Körperschaften [29], sind gemeinsam von der Gesetzgebung, Krankenversicherung, Patientenvertretung, Wissenschaft und Körperschaften zu veranlassen. Hier sollte zukünftig besonders die Versorgung in der Häuslichkeit im Vordergrund stehen, in der die meisten SmP leben. Sie entwickeln hier oft ein beschwerdeorientiertes Inanspruchnahmeverhalten, dessen negative Folgen oft erst in einer anschließenden stationären Pflegesituation auffallen.

**Table 5:** 

Merkmale der aufsuchenden zahnmedizinischen Betreuung	Umsetzung mit geringem Aufwand	Umsetzung mit mittlerem Aufwand	Umsetzung mit hohem Aufwand
Screening/Befundaufnahme	Durchführen	Durchführen	Durchführen
Behandlungen	Keine	Einfache	(Fast) alle
Ausstattung	Grundbesteck^a^	Grundbesteck^a^	Grundbesteck^a^
	Fahrzeug	Fahrzeug	Fahrzeug
		Instrumente und Materialien entsprechend den Behandlungen	Instrumente und Materialien entsprechend den Behandlungen
		Kleine mobile Behandlungseinheit	Kleine mobile Behandlungseinheit
		Mobiles Ultraschallgerät	Oder praxisübliche zahnärztliche Behandlungseinheit
			Evtl. zahnärztlicher mobiler Behandlungsstuhl
			Mobiles Ultraschallgerät
Ort der Röntgendiagnostik	Praxis	Praxis, selten mobil	Mobil
Ort der Behandlung bei einfachen Interventionen, z. B. einfachen Extraktionen	Praxis	Pflegeeinrichtung	Pflegeeinrichtung
		Häuslichkeit	Häuslichkeit
Ort der Behandlung bei schwierigen Interventionen, z. B. bei mehreren oder schwierigen Extraktionen	Praxis	Praxis	Pflegeeinrichtung
			Häuslichkeit
			(Selten Praxis)

^a^Grundbesteck = Spiegel, Sonden, Pinzette, Serviette, Watterollen, Handschuhe, Desinfektionstücher, Schreibunterlagen etc.

### Vernetzung ambulanter und stationärer Krankenhausversorgung

Eine weitere Herausforderung besteht in der Verbesserung der Vernetzung von ambulanten und stationären Versorgungsstrukturen.

#### Kliniken für Geriatrie

Während der Behandlung in den Kliniken für Geriatrie werden fast alle Körperteile und deren Funktionen untersucht, ggf. Therapiekonzepte erstellt und umgesetzt. Die Mundhöhle ist dabei jedoch fast immer ausgeschlossen, da Studierende der Humanmedizin während ihres Studiums nur wenig Berührungspunkte mit zahnmedizinischen Krankheitsbildern haben. Erkrankungen der Mundhöhle werden somit selten diagnostiziert. Zahnärztinnen und Zahnärzte sind in die stationäre geriatrische Versorgung nicht eingebunden [30–35]. Hauptgrund für diesen Sachverhalt ist, dass die erbrachten zahnärztlichen Leistungen bei einer Behandlung im stationären Setting nicht wie üblich über die kassenzahnärztliche Vereinigung mit der gesetzlichen Krankenkasse abgerechnet werden. Dies führt dazu, dass in aller Regel nur Notfallversorgungen stattfinden. Da zahnärztliche Behandlungen nicht im stationären Abrechnungssystem abgedeckt sind und die abgebildeten kieferchirurgischen Leistungen nicht denjenigen entsprechen, die zur Versorgung von Patienten in den akutgeriatrischen Stationen benötigt werden, verstreicht häufig die Gelegenheit, geriatrische Patientinnen und Patienten auch über ihre Mundgesundheit aufzuklären, einen Weg zur wohnortnahen zahnärztlichen Behandlung anzubahnen und ggf. kleinere Maßnahmen mobil ausgerüstet sofort im Krankenhaus durchzuführen. Dies ist bedauerlich, bestünde doch beispielsweise die Chance, schwierige Extraktionen unter Antikoagulation mit ärztlicher Unterstützung während des Krankenhausaufenthaltes vorzunehmen und damit auch spätere kostenintensive Transporte zum Zahnarzt zu reduzieren. Es sollte demnach ein Weg gefunden werden, dass zahnärztliche Leistungen bei stationär aufgenommenen geriatrischen Patientinnen und Patienten mit den gesetzlichen Krankenversicherern abgerechnet werden können.

#### Zahnärztliche Behandlungen bei Abwehrverhalten von Patientinnen und Patienten

Zahnärztinnen und Zahnärzte können der Herausforderung gegenüberstehen, dass eine Behandlung verbal oder schlussendlich auch durch Abwehrhandlungen verweigert wird. Besonders problematisch wird es z. B., wenn ein ehemals sanftmütiger Patient plötzlich ausgeprägt jähzornig wird, weil eine demenzielle Erkrankung Aggressionen mit sich bringt. Angst tritt dann als Grund für Wutanfälle in Erscheinung, wobei Menschen mit Demenz ein erhöhtes Risiko haben, sich selbst und andere zu gefährden [24, 36]. Zahnärztinnen und Zahnärzte, Angehörige und Pflegende sind hier in einer schwierigen Situation: Einerseits dürfen sie die Freiheitsrechte der betroffenen Person nicht unnötig einschränken, andererseits müssen sie zu deren Wohl handeln und dafür sorgen, dass die Mundpflege und zahnärztliche Behandlung möglichst aufrechterhalten bleibt. Freiheitseinschränkende Maßnahmen wie das Festhalten der betroffenen Person dürfen im Rahmen der Behandlung nur dann eingesetzt werden, nachdem alle anderen Möglichkeiten ausgeschöpft sind, um die Gefahr für die zu Behandelnden oder für Dritte zu verringern. Ferner ist zu berücksichtigen, dass eine zahnärztliche Behandlung einer festgehaltenen Person schon durch kleinste Bewegungen des Kopfes und die rotierenden Geräte im Mund ohne Verletzungen kaum möglich ist. Nur wenn das Risiko der Eigengefährdung oder der Gefährdung Dritter mit anderen Mitteln nicht in ausreichendem Maße vermeidbar erscheint, sollte eine zahnärztliche Behandlung in Intubationsnarkose (ITN) erfolgen [37, 38]. In der Regel bestehen Probleme bei der Umsetzung einer ambulanten Anästhesie für orale Sanierungen (z. B. Ort, Vergütung). Zunehmend sind Menschen mit Demenz und/oder mit zusätzlicher ausgeprägter Multimorbidität nicht mehr im Rahmen einer ambulanten Versorgung unter Sedierung oder ITN zahnärztlich zu versorgen. Vonseiten der Anästhesie kann oftmals eine etwaig auftretende Notfallsituation mit anschließender stationärer Behandlung und Beobachtung nicht ausgeschlossen werden. Dabei fällt eine stationäre Versorgungslücke für diese zahnärztlichen Behandlungen auf, wobei diese mit fachgerechter Nachsorge in den Kliniken für Geriatrie integriert werden könnten.

### Finanzierung des Mehraufwandes der zahnmedizinischen Betreuung

Es ist eine Herausforderung, ältere Menschen mit Multimorbidität und Polymedikation zahnmedizinisch zu betreuen. Dafür sind zusätzliche Kenntnisse notwendig, die während des Studiums der Zahnmedizin gegenwärtig kaum adäquat gelehrt werden (können; [39]). Die wissenschaftliche Fachgesellschaft (Deutsche Gesellschaft für Alterszahnmedizin e. V.) bietet daher eine Spezialisierung in Seniorenzahnmedizin an [40], da die Etablierung und Koordination einer zahnmedizinischen Betreuung unter Einbindung des Praxisteams, der hausärztlichen Versorgung, des Pflegepersonals und der Angehörigen bei zunehmender Gebrechlichkeit komplex sind. Generell sind die zahnärztlichen Behandlungen zu den gleichen Bedingungen abrechenbar wie bei allgemeinmedizinisch gesunden Personen, obwohl der zeitliche Mehraufwand oftmals deutlich höher ist. Dies ist auch unter dem Umstand zu bewerten, dass SmP aufgrund ihrer Multimorbidität meist nicht mehr selbstständig regelmäßig eine zahnärztliche Praxis aufsuchen können. Zwischenzeitlich wurden bereits einige Verbesserungen hinsichtlich der Honorierung derartiger zahnärztlicher Leistungen geschaffen [26–28]. Diese reichen jedoch bis dato nicht aus, um eine genügende Anzahl an Zahnmedizinerinnen und -medizinern zu motivieren, auch für SmP auf fachlich hohem Niveau zur Verfügung zu stehen. Der Mehraufwand für die einzelnen zahnärztlichen Leistungen ist bisher nicht berücksichtigt. Dies unterstreicht, dass auf verschiedenen Ebenen die Voraussetzungen für eine verbesserte Inanspruchnahme zahnärztlicher Leistungen geschaffen werden sollten.

## Zahnmedizinisches Problembewusstsein in der Pflege

### Allgemeine Situation

Studien zeigen, dass das Pflegepersonal die Herausforderungen der Mund- und Prothesenpflege aus Zeitgründen und aufgrund fehlenden theoretischen sowie praktischen Wissens nicht ausreichend erfüllen kann [21, 41–43]. In diesem Kontext sollten verschiedene Anknüpfungspunkte zwischen Pflege und Zahnmedizin in Aus‑, Fort- und Weiterbildung weiterentwickelt werden [23, 44–46]. Die durch die gesetzliche Krankenversicherung finanzierten Kooperationsverträge zwischen zahnärztlichen Praxen und Pflegeeinrichtungen [47] ermöglichen den Zahnmedizinerinnen und -medizinern eine bessere Kommunikation und Darstellung der Mundsituation gegenüber der Pflege. In diesem Zusammenhang ist eine Vergütung für die Zahnmedizinerinnen und -mediziner, nicht aber für die Pflege vorhanden.

### Eine Chance: Expertenstandard Mundgesundheit

Das Deutsche Netzwerk für Qualitätsentwicklung in der Pflege (DNQP) entwickelt seit 20 Jahren Expertenstandards (ES), die professionsübergreifend als ein anerkanntes Instrument zur Qualitätsentwicklung in der Pflege gelten [48]. Seit 2019 wird in Kooperation mit der Bundeszahnärztekammer, der Deutschen Gesellschaft für Alterszahnmedizin und der Arbeitsgemeinschaft Zahnmedizin für Menschen mit Behinderung oder besonderem medizinischen Unterstützungsbedarf ein neuer ES zur „Förderung der Mundgesundheit in der Pflege“ entwickelt. Im Gegensatz zu den anderen ES ist neben der Profession der Pflege erstmalig auch eine weitere Berufsgruppe, die Zahnmedizinerinnen und -mediziner, am Entwicklungsprozess beteiligt. Mit der Professionalisierung der Pflege steigt die Nachfrage nach ES kontinuierlich. Zusätzlich sind ES ein Bezugspunkt für die Qualitätsbeurteilung von Pflegeeinrichtungen durch den Medizinischen Dienst (Sozialgesetzbuch (SGB), elftes Buch (XI) – Soziale Pflegeversicherung; [49]). ES konkretisieren das aktuelle Wissen, wurden in der Vergangenheit von Gerichten herangezogen und haben auch für den Bereich des SGB, fünftes Buch (V) – Gesetzliche Krankenversicherung Relevanz. Es ist davon auszugehen, dass sich die Aufmerksamkeit der Pflege für Probleme in der Mundhöhle und der Mundpflege mit der Einführung des ES erhöhen wird. Klare Beschreibungen im Hinblick auf die Struktur‑, Prozess- und Ergebnisqualität in der Pflege werden eine sehr gute Orientierung sowohl im Hinblick auf die interne Qualitätsentwicklung als auch auf die externe Qualitätssicherung bewirken. Der ES zur „Förderung der Mundgesundheit in der Pflege“ wird in die Ausbildung (auch Bachelor- und Masterstudiengänge) sowie Fort- und Weiterbildung im Pflegebereich einfließen. Die Zahnärztinnen und Zahnärzte sollten die Implementierung dieses ES als Chance wahrnehmen und seine Umsetzung unterstützen.

## Fazit

Die zahnmedizinische Versorgung älterer Menschen steht vor der Herausforderung, dass sie zum Teil über Jahrzehnte sehr heterogene Lebensphasen mit unterschiedlichen zahnmedizinischen Ansprüchen begleiten muss. In der fitten Lebensphase gilt es, präventivzahnmedizinische Aufgaben und eine gesunde Mundhöhle sicherzustellen. In der gebrechlichen Lebensphase ist das Ziel, idealerweise zusammen mit der Hilfe von Angehörigen eine Mundsituation zu schaffen, die stabil und leicht zu pflegen ist. Diese Phase sollte engmaschig präventiv begleitet werden. Mit dem Auftreten von Pflegebedürftigkeit sind für die Zahnärztin oder den Zahnarzt Ansprechpersonen zu etablieren, die mit Fachwissen die in den früheren Phasen hergestellte gute Mundsituation erhalten. Es ist wichtig, dass alle Akteure im Gesundheitswesen auch für die heterogene Gruppe der Seniorinnen und Senioren einen möglichst chancengleichen und barrierearmen Zugang zu einer zahnmedizinischen Versorgung schaffen. Das Gesundheitswesen mit seinen Akteuren in den Makro‑, Meso- und Mikroebenen und seinen Strukturen sowie Schnittstellen ist gefordert, nicht nur eine zahnärztliche Behandlung, sondern eine zahnmedizinische Betreuung bereitzustellen, damit auch bei schwierigen Lebensumständen eine bestmögliche Mundgesundheit erreicht wird. Neben einer besseren Vernetzung ambulanter und stationärer Strukturen ist es eine Herausforderung, das Problembewusstsein innerhalb und außerhalb der zahnmedizinischen Berufsgruppen zu stärken und mundgesundheitsbezogene Aus‑, Fort- und Weiterbildungsangebote zu etablieren.
